# GNSS Space-Time Interference Mitigation and Attitude Determination in the Presence of Interference Signals

**DOI:** 10.3390/s150612180

**Published:** 2015-05-26

**Authors:** Saeed Daneshmand, Ali Jafarnia Jahromi, Ali Broumandan, Gérard Lachapelle

**Affiliations:** PLAN Group, Department of Geomatics Engineering, Schulich School of Engineering, University of Calgary, 2500 University Drive, N.W., Calgary, AB T2N 1N4, Canada; E-Mails: ajafarni@ucalgary.ca (A.J.J.); abrouman@ucalgary.ca (A.B.); gerard.lachapelle@ucalgary.ca (G.L.)

**Keywords:** antenna array receiver, global navigation satellite system (GNSS), interference mitigation, space-time processing

## Abstract

The use of Space-Time Processing (STP) in Global Navigation Satellite System (GNSS) applications is gaining significant attention due to its effectiveness for both narrowband and wideband interference suppression. However, the resulting distortion and bias on the cross correlation functions due to space-time filtering is a major limitation of this technique. Employing the steering vector of the GNSS signals in the filter structure can significantly reduce the distortion on cross correlation functions and lead to more accurate pseudorange measurements. This paper proposes a two-stage interference mitigation approach in which the first stage estimates an interference-free subspace before the acquisition and tracking phases and projects all received signals into this subspace. The next stage estimates array attitude parameters based on detecting and employing GNSS signals that are less distorted due to the projection process. Attitude parameters enable the receiver to estimate the steering vector of each satellite signal and use it in the novel distortionless STP filter to significantly reduce distortion and maximize Signal-to-Noise Ratio (SNR). GPS signals were collected using a six-element antenna array under open sky conditions to first calibrate the antenna array. Simulated interfering signals were then added to the digitized samples in software to verify the applicability of the proposed receiver structure and assess its performance for several interference scenarios.

## 1. Introduction

GNSS is used worldwide, however, the performance of location-based services provided by receivers can still be compromised by interfering signals. There is an ever increasing attention to safe and secure applications. As an example, countless time tagging and synchronization systems in the telecom and electrical power grid industries relying primarily on GNSS signals are vulnerable to in-band electronic interference because of being extremely weak signals broadcasted over wireless channels. Therefore, even low-power interference can easily jam receivers within a radius of several km. Interference can dramatically degrade the performance of receivers or completely deny position and time services provided by these systems. The spread spectrum modulation technique applied in the structure of GNSS signals provides a certain degree of protection against narrowband interfering signals; however, the spreading gain alone is not sufficient to suppress high power or wideband interference signals.

Over the years, GNSS interference suppression methods based on time and frequency processing have been widely studied in the literature (e.g., [1,2]). Although they are effective for suppressing continuous wave (CW) or multi-tone jammers, their performance degrades when they deal with wideband interference signals (e.g., Gaussian jammers) or when interfering signals change rapidly in time and frequency such as swept continuous wave interference [3].

On the contrary, interference mitigation techniques utilizing an antenna array can effectively detect and suppress both narrowband and wideband interfering signals regardless of their time and frequency characteristics. One of the earliest space-based processing methods has been referred to as the Capon beamformer, also called minimum variance distortionless response (MVDR) beamformer [4,5]. The MVDR beamformer has a distortionless response for the desired signal while suppressing all signals arriving from other directions. Antenna array processing in GNSS applications has been mostly centered on interference suppression [6,7,8,9,10]. Reference [10] drew the attention on utilizing minimum power distortionless response (MPDR) beamforming for GPS applications to reject interference signals whose power is significantly higher than that of the GPS signals. MPDR and MVDR beamformers have been considered popular methods for a variety of signal processing applications such as radar, wireless communications, and speech enhancement.

Despite the effectiveness of antenna array-based methods, they suffer from hardware complexity. In spatial processing, the number of antennas determines the number of interfering signals that can be suppressed. Limitations on the number of the antennas and size of the array can be considered the main practical challenge of these methods. To deal with this problem, techniques employing both time/frequency and spatial domain processing have been of great interest since, in contrast to time/frequency based methods, they are able to deal with both wideband and narrowband interference. These techniques are generally referred to as Space-Time Adaptive Processing (STAP) or Space-Frequency Adaptive Processing (SFAP) techniques. STAP and SFAP approaches in GNSS applications have been studied in the literature for several years [11,12,13,14,15]. These methods combine spatial and temporal filters to suppress more radio frequency interfering signals by increasing the Degree of Freedom (DoF) of the array without physically increasing the antenna array size. The term “adaptive” means that the array follows changes in environment and constantly adapts its own pattern by means of a feedback control. The main focus here is on space-time processing and the study of adaptive methods is not considered. Nevertheless, the proposed approach can be easily extended to the adaptive cases as well.

Besides the superior advantages of space-time filtering, specific considerations should be taken into account in designing such filters in order to prevent induced biases in pseudorange measurements and achieve accurate and precise time and position solutions [16,17]. The output of the space-time filter is basically a direction-frequency dependent response. Even if the filter completely nullifies interfering signals, the non-linearity behavior of its frequency response may result in biased measurements, distortion or broadness of the cross correlation functions during receiver acquisition and tracking stages. This may not be tolerable especially for high precision GNSS applications. The effects of this distortion on GNSS signals were recently studied [16,17,18,19].

To reduce this distortion, one effective approach is to incorporate the satellite signal steering vector, which contains all the spatial information of the incoming signal, in the structure of the space-time filter as a constrained optimization problem [19,20,21,22,23]. In fact, these methods are extended versions of MVDR and MPDR beamformers for space-time processing. Although employing the satellite steering vectors in the beamformer structure avoids unintentional signal attenuation, the resulting cross correlation functions after beamforming may still be distorted, which in turn produces biases on pseudorange measurements and errors in the position solutions. This is due to the fact that in these techniques there is no explicit assumption on the linearity of the STP filter response. In [9], it is suggested that the space-time filter be designed to have a real frequency response (formed from a filter multiplied by its conjugate); however this was not analyzed and a practical realization of filter coefficients was not reported. There are other effective approaches to reduce the induced bias error; however, they do not guarantee a distortionless response for GNSS signals [16,18].

Although employing the satellite signal steering vector has been widely employed in the STP processing, limited papers addresses the steering vector estimation in the presence of interference. The steering vector conveys the spatial information that can be also employed for various applications such as multipath mitigation, SNR maximization and Angle of Arrival (AOA) estimation. Steering vector estimation in a jammed environment for attitude determination was studied in [24]; in this paper it is assumed that the spatial covariance matrix is positive definite and invertible which may not be the case in all inference scenarios where the covariance matrix becomes ill-conditioned.

The steering vector can be obtained by either measuring phase differences of the received signals at the antenna array elements, or by calculating it from array platform attitude parameters and satellite azimuth and elevation angles. The first approach needs to acquire and track the received signals, some of which may not be available in challenging environments. The second approach can calculate the steering vector of all satellites regardless of the signal quality and availability but requires attitude parameters. Herein both methods are employed in the proposed structure of the receiver such that steering vectors, measured from the available satellite signals, are used to estimate attitude parameters and consequently the steering vector of all satellite signals without imposing any assumption on the spatial covariance matrix.

The other part of this research focuses on space-time filter design for GNSS interference mitigation. Due to the simplicity in implementation, Space-Time Processing (STP) filters in GNSS applications are mostly implemented before the despreading process (*i.e.*, correlation and Doppler removal). However, since satellite steering vectors are not employed in the structure of the filter, some satellite signals may be attenuated or distorted, which will adversely affect the performance of the receiver. Therefore, herein a two-stage receiver structure is proposed. The first stage is implemented before the despreading process. In this stage, by estimating a projection matrix from the Singular Value Decomposition (SVD) of the pre-despreading space-time covariance matrix, the received signals are projected into the interference-free subspace. Estimating the signal steering vector from the projected subspace is an underdetermined problem and it is shown that extracting the spatial information from the projected signals may not be possible or can be partially done for some signals. In fact, some of the array DOF information is used to remove interfering signals. Therefore, in the second stage, attitude parameters are estimated considering those steering vectors that could be estimated. By using attitude parameters and satellite azimuth and elevation angles from ephemeris data, the steering vector of all satellite signals can be then accurately calculated and employed in designing a space-time filter. A novel approach for designing the space-time filter is also proposed not only to nullify the interference signals but also to increase the C/N_0_ and to avoid biases and distortions on cross correlation functions.

In order to verify the effectiveness of the proposed method for steering vector estimation in the presence of interference and assess its performance, a set of real GPS L1 signals was collected and simulated interfering signals were added to the digitized samples in software. A tactical-grade IMU was used as reference to evaluate the accuracy of satellite steering vectors and heading estimates. Moreover, to evaluate the performance of the proposed space-time filter, it was compared to the well-known space-only MPDR and space-time MPDR beamformer methods.

## 2. Signal Model

**Figure 1:**
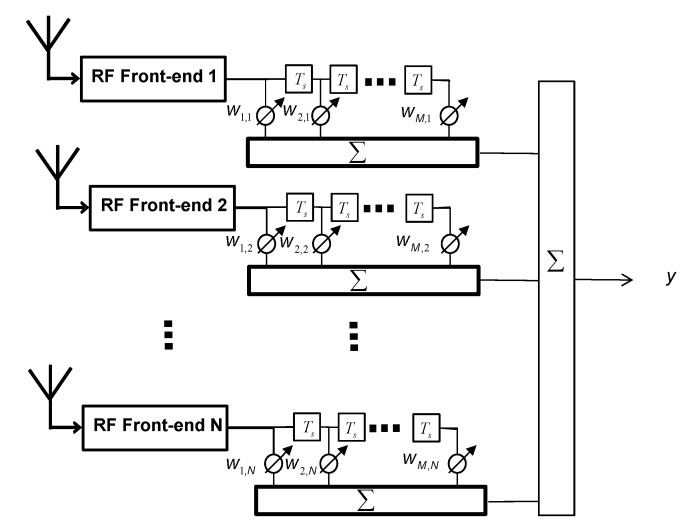
Generic structure of a space-time filter.

Without loss of generality and for the sake of simplicity, only one GNSS signal is considered in the formulations below. Complex baseband representation of the received signal vector at an arbitrary *N*-element array configuration for the satellite signal and *K* interference signals can be written as:

(1) $$\underset{N\times 1}{r}=\underset{N\times 1}{a}s+\underset{N\times K}{B}\underset{K\times 1}{v}+\underset{N\times 1}{\eta }\;$$

where **B** is a matrix whose columns indicate interfering signal steering vectors and **η** is a complex additive white Gaussian noise vector. *s* represents the GNSS signal waveform and **v** is a vector specifying *K* interfering signal waveforms. In Equation (1), **a** is an *N* × 1 vector representing the steering vector (or array manifold vector) of the satellite signal defined as:

(2) $$a≜\left[\begin{array}{c} e^{j\frac{2\pi }{\lambda }{\left(d\right)}^{T}z_{1}}\\ e^{j\frac{2\pi }{\lambda }{\left(d\right)}^{T}z_{2}}\\ \vdots \\ e^{j\frac{2\pi }{\lambda }{\left(d\right)}^{T}z_{N}} \end{array}\right]$$

in which λ is the wavelength of the signal and ***z****_n_*, *n* = 1, 2, …, *N* is a 3 × 1 unit vector pointing to the *n*th antenna element and **d** is a 3 × 1 unit vector pointing to the satellite direction in the body frame coordinate system and *T* stands for the transpose operation. Figure 1 shows that the standard implementation of the STP filter in which each antenna is followed by a temporal filter or a Tapped Delay Line (TDL) with the typical delay time of a sampling duration denoted by *T_s_*.

An antenna array with *N* elements and TDLs with *M* − 1 taps leaves *MN* unknown filter coefficients which should be determined. For each time snapshot, *MN* received samples form a *MN* × 1 vector can be written as:

(3) $$\vec{r}={\left[\begin{array}{cccccccccccc} r_{1,1} & r_{1,2} & \cdots & r_{1,N} & r_{2,1} & r_{2,2} & \cdots & r_{2,N} & \cdots & r_{M,1} & \begin{array}{c} r_{M,2} \end{array} & \cdots \end{array}\;\begin{array}{c} r_{M,N} \end{array}\right]}^{T}$$

in which *r_m_*_,*n*_ is the *m*th delayed sample at the *n*th antenna element. Filter coefficients corresponding to these samples are defined as:

(4) $$\vec{w}={\left[\begin{array}{cccccccccccc} w_{1,1} & w_{1,2} & \cdots & w_{1,N} & w_{2,1} & w_{2,2} & \cdots & w_{2,N} & \cdots & w_{M,1} & \begin{array}{c} w_{M,2} \end{array} & \cdots \end{array}\;\begin{array}{c} w_{M,N} \end{array}\right]}^{H}$$

Hence, the space-time filter output is obtained as:

(5) $$y={\vec{w}}^{H}\vec{r}$$

in which *H* denotes the conjugate transpose. In order to suppress high power interference, power of the filter output should be minimized as:

(6) $$\underset{\left\|\vec{w}\right\|=1}{\min}\;E\left\{{\left\|y\right\|}^{2}\right\}={\vec{w}}^{H}R_{\vec{r}}\vec{w}$$

where *E*{} represents the statistical expectation and $R_{\vec{r}}$ is the spatial-temporal covariance or correlation matrix defined as:

(7) $$\underset{MN\times MN}{R_{\vec{r}}}=E\left\{\vec{r}{\vec{r}}^{H}\right\}$$

In the following section, a projection matrix into the interference-free subspace is calculated based on this correlation matrix in order to mitigate interference.

## 3. Proposed Receiver Structure

**Figure 2:**
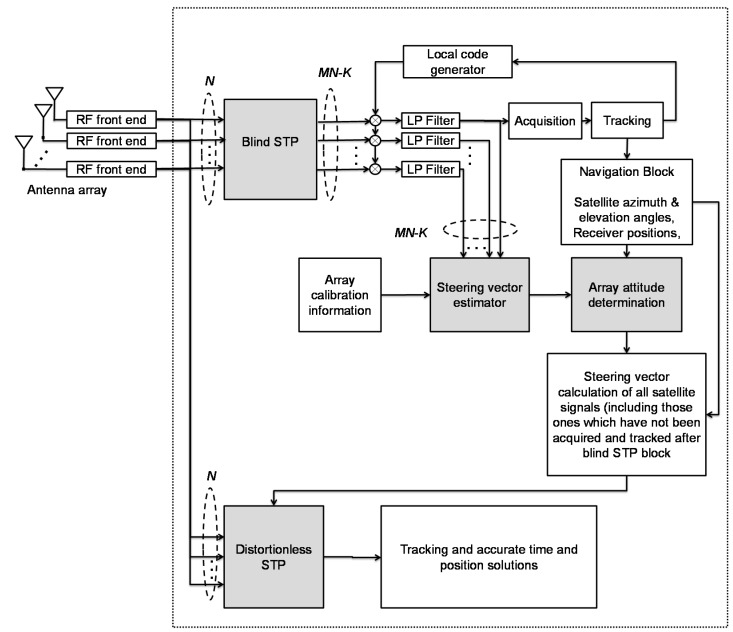
Structure of the proposed receiver.

The proposed receiver structure incorporates two interference suppression modes namely blind and distortionless and is shown in Figure 2.

The blind STP block calculates a projection matrix to the interference-free subspace from spatial temporal down-converted samples. Each row of the projection matrix provides a set of space-time filter coefficients. Assigning all these sets to the corresponding number of the space-time filters results in several interference-free outputs. Afterwards, only the satellite signals at one of these outputs are acquired and tracked, and the locally generated codes are used to despread the signals at other outputs. The steering vector estimator block employs the output of the blind STP block and pre-determined array calibration information to estimate the projected component of the steering vector of available satellite signals into the interference-free subspace. This enables the attitude determination block to calculate the array attitude parameters. By having attitude parameters, satellite azimuth and elevation angles, the steering vector of all satellites before projection (even if these are not initially acquired and tracked) can be accurately estimated. This information is then employed in the distortionless STP block. This block assigns different filter coefficients for different satellite signals to maintain the main lobe of the beam pattern in the direction of each signal and to decrease the induced distortions and biases on pseudorange measurements. In the following subsections, four processing blocks namely “Blind STP”, “Distortionless STP”, “Steering vector estimator” and “Attitude determination” are described. For the sake of simplicity in problem formulation, the organization of these subsections is based on the processing level in the receiver rather than the data flow in the receiver structure.

### 3.1. Blind STP

Here the projection to an interference-free subspace is referred to as a blind method since satellite signal steering vectors are not considered in the filter design. In order to be destructive at correlator outputs, the power of the interference signal should be significantly higher than that of the GNSS and noise signals. This makes the interference subspace easily distinguished and estimated. A projection matrix into the interference-free subspace can be estimated by performing an Eigenvalue Decomposition (EVD) or Singular Value Decomposition (SVD) of $R_{\vec{r}}$ as:

(8) $$R_{\vec{r}}=\left[\begin{array}{cc} \underset{MN\times K}{U_{Int}} & \underset{MN\times (MN-K)}{U_{Null}} \end{array}\right]\left[\begin{array}{cc} \underset{K\times K}{\Lambda_{Int}} & 0\\ 0 & \underset{\left(MN-K)\times (MN-K\right)}{\Lambda_{Null}} \end{array}\right]\left[\begin{array}{c} {U_{Int}}^{H}\\ {U_{Null}}^{H} \end{array}\right]\;$$

where **U***_int_* and **U***_null_* are eigenvector matrices of interference and the noise-plus-GNSS signal subspaces, respectively, and **Λ***_int_* and **Λ***_Null_* are corresponding eigenvalue matrices. In Equation (8), *K* indicates the rank of the interference subspace (without loss of generality, it is assumed that interfering signals are uncorrelated). Hence, a projection matrix into the reduced-rank interference-free subspace can be calculated as $U_{Null}^{H}$ which is formed from *MN* − *K* eigenvectors corresponding to the *MN* − *K* smallest eigenvalues. In fact the filter gain vector $\vec{w}$, which minimizes the filter output power in Equation (6), belongs to this interference-free subspace. Applying this projection matrix to the received signal vector suppresses the interference; however, some satellite signals may become distorted or attenuated in the process.

### 3.2. Distortionless STP

The output of the blind space-time filter is basically a direction-frequency dependent response. Even if the filter completely nullifies interfering signals, the non-linearity behavior of its frequency response may result in distorted correlation functions, degraded acquisition and tracking performance, and biases on the GNSS measurements. In addition, since the spatial information of the satellite signals is not considered in the blind beamforming process, some signals may be unintentionally attenuated due to the nulls in the beam pattern. This may not be tolerable especially for high precision applications.

A novel approach for designing a distortionless space-time filter for interference mitigation is therefore proposed such that the filter impulse response becomes symmetric and linear in phase, and therefore the same delay is added to all pseudoranges. The proposed distortionless filter is based on subspace decomposition and incorporating the knowledge of the steering vectors. This section first introduces a possible structure for distortionless space-time filter and then estimates corresponding filter coefficients not only to mitigate interference signals but also to satisfy the distortionless condition.

For each satellite signal, the filter coefficients should be assigned differently, which is commensurate to the steering vector of the signal; however the structure of the filter remains the same. Therefore, without loss of generality, one GNSS signal is considered in the formulations. Assume that the power spectrum of the signal after despreading is defined as *p*(*f*) and ***h***(*f*) is the *N* × 1 frequency response vector of the space-time filter. The correlation function for the signal after space-time filtering, despreading and Doppler removal can be written as [9]:

(9) $$R\left(\tau ,a\right)=\int_{-\infty }^{\infty }h^{H}\left(f\right)ap\left(f\right)e^{-j2\pi f\tau }df$$

where **a** is the steering vector defined in Equation (2). *p*(*f*) is a symmetric function of frequency and therefore in the absence of **h***^H^*(*f*)**a**, the correlation function has a peak at τ. In order to avoid bias and to have a symmetric correlation function, **h***^H^*(*f*)**a** should have a symmetric conjugate impulse response. This results in a linear phase space-time filter and consequently a symmetrically broadened correlation function for each satellite signal with a bias that is similar for all signals. Therefore, all pseudorange measurements are affected in the same way and position performance is not affected. The only performance degradation ance may be in tracking due to the broadness of the correlation functions. In this paper, the following structure for the distortionless STP is proposed:

(10) $$h\left(f\right)=\sum_{i=1}^{M}AP_{\left(i\right)}^{H}ue^{j2\pi ifT_{s}}$$

where **A** is a diagonal matrix whose elements are equivalent to those of the steering vector and **u** is a vector weighting the rows of the projection matrix and $P_{\left(i\right)},i=1,2,..M$ can be obtained by partitioning the projection matrix of interest **P** as:

(11) $$P=\left[\begin{array}{cccc} P_{\left(1\right)}A^{H} & P_{\left(2\right)}A^{H} & \cdots & P_{\left(M\right)}A^{H} \end{array}\right]\;$$

**P** and **u** should be determined to hold the following conditions:

(1) Considering Equation (11), **P** should suppress the interfering signals and has the following structure:
  (12) $$\{\begin{array}{c} \;M\;odd\;\left\{ \begin{array}{l} P_{\left(i\right)}={\left(P_{\left(M-i+1\right)}\right)}^{*}\;,\;i=1,\;2,...,\frac{M-1}{2}\\ P_{\left(\frac{M+1}{2}\right)}\;is\;real \end{array}\right.\\ \;M\;even\;P_{\left(i\right)}={\left(P_{\left(M-i+1\right)}\right)}^{*}\;,\;i=1,\;2...,\frac{M}{2}\; \end{array}\;$$
  where ()* denotes conjugate operation.
(2) Vector **u** is determined such as to maximize the correlation function and consequently maximize the SNR of the projected satellite signal.

This structure coerces the filter coefficients for each TDL to be conjugate symmetric and therefore to be a linear phase. Moreover, by applying matrix **A** in the projection matrix, the filter compensates for spatial phase differences of the received signal among antenna elements and consequently the resulting space-time filter from the combination of the linear phase TDLs remains linear phase and the cross correlation function is symmetrically broadened. In order to design the projection matrix in the form of Equation (12), the filter output is calculated as:

(13) $$y={\vec{w}}^{H}A^{H}\vec{r}$$

in which the following condition on the filter coefficients is applied:

(14) $$\;w_{m,n}={\left(w_{M-m+1,n}\right)}^{*},\;m=1,...,\frac{M}{2}\;$$

and A is a block diagonal matrix defined as:

(15) $$\underset{NM\times NM}{A}=\left[\begin{array}{cccc} A & 0 & \cdots & \underset{N\times N}{0}\\ 0 & A & \cdots & 0\\ \vdots & \vdots & \ddots & \vdots \\ 0 & 0 & \cdots & A \end{array}\right]$$

In Equation (14), *M* is assumed even. The process can be easily repeated with a few modifications when *M* is odd. Considering Equation (14), half of the filter coefficients are related to each other and only half of the coefficients should be determined. In fact the DoF is halved to constrain the filter structure. The filter output in Equation (13) can be spilt into the sum of two terms as:

(16) $$y={\vec{w}}_{s}^{H}{\bar{A}}^{H}{\vec{r}}_{1}+{\left({\vec{w}}_{s}^{H}\bar{A}{\vec{r}}_{2}\right)}^{H}$$

where:

(17) $$\begin{array}{l} {\vec{r}}_{1}={\left[\begin{array}{cccccccccccc} r_{1,1} & r_{1,2} & \cdots & r_{1,N} & r_{2,1} & r_{2,2} & \cdots & r_{2,N} & \cdots & r_{\frac{M}{2},1} & \begin{array}{c} r_{\frac{M}{2},2} \end{array} & \cdots \end{array}\;\begin{array}{c} r_{\frac{M}{2},N} \end{array}\right]}^{T}\\ {\vec{r}}_{2}={\left[\begin{array}{cccccccccccc} r_{M,1}^{*} & r_{M,2}^{*} & \cdots & r_{M,N}^{*} & r_{M-1,1}^{*} & r_{M-1,2}^{*} & \cdots & r_{M-1,N}^{*} & \cdots & r_{\frac{M}{2}+1,1}^{*} & \begin{array}{c} r_{\frac{M}{2}+1,2}^{*} \end{array} & \cdots \end{array}\;r_{\frac{M}{2}+1,N}^{*}\right]}^{T}\\ {\vec{w}}_{s}={\left[\begin{array}{cccccccccccc} w_{1,1} & w_{1,2} & \cdots & w_{1,N} & w_{2,1} & w_{2,2} & \cdots & w_{2,N} & \cdots & w_{\frac{M}{2},1} & \begin{array}{c} w_{\frac{M}{2},2} \end{array} & \cdots \end{array}\;\begin{array}{c} w_{\frac{M}{2},N} \end{array}\right]}^{H}\\ \underset{N\frac{M}{2}\times N\frac{M}{2}}{\bar{A}}=\left[\begin{array}{cccc} A & 0 & \cdots & \underset{N\times N}{0}\\ 0 & A & \cdots & 0\\ \vdots & \vdots & \ddots & \vdots \\ 0 & 0 & \cdots & A \end{array}\right] \end{array}$$

In order to suppress interference, ${\vec{w}}_{s}^{H}$ should be determined so that the filter output power is minimized. To calculate a closed form solution for ${\vec{w}}_{s}^{H}$, instead of minimizing the total power, the sum of power of each term in Equation (16) is minimized. This sub-optimization is expressed as:

(18) $$\underset{\left\|{\vec{w}}_{s}\right\|=1}{\min}\;E\left\{{\left\|{\vec{w}}_{s}^{H}{\bar{A}}^{H}{\vec{r}}_{1}\right\|}^{2}+{\left\|{\vec{w}}_{s}^{H}\bar{A}{\vec{r}}_{2}\right\|}^{2}\right\}$$

The optimization in Equation (18) can be written as:

(19) $$\underset{\left\|{\vec{w}}_{s}\right\|=1}{\min}\;{\vec{w}}_{s}^{H}\;\left({\bar{A}}^{H}R_{{\vec{r}}_{1}}\bar{A}+\bar{A}R_{{\vec{r}}_{2}{\vec{r}}_{1}}{\bar{A}}^{H}\right){\vec{w}}_{s}$$

in which:

(20) $$\begin{array}{l} \underset{\frac{M}{2}N\times \frac{M}{2}N}{R_{{\vec{r}}_{1}}}=E\left\{{\vec{r}}_{1}{{\vec{r}}_{1}}^{H}\right\}\\ \underset{\frac{M}{2}N\times \frac{M}{2}N}{R_{{\vec{r}}_{2}}}=E\left\{{\vec{r}}_{2}{{\vec{r}}_{2}}^{H}\right\} \end{array}$$

From Equation (19), it can be concluded that the projection matrix to the interference-free subspace (${\vec{w}}_{s}$ belongs to this subspace) can be calculated as the following SVD problem:

(21) $${\bar{A}}^{H}R_{{\vec{r}}_{1}}\bar{A}+\bar{A}R_{{\vec{r}}_{2}}{\bar{A}}^{H}=\left[\begin{array}{cc} U & V \end{array}\right]\left[\begin{array}{cc} \Lambda_{Int} & 0\\ 0 & \Lambda_{Null} \end{array}\right]\left[\begin{array}{c} U^{H}\\ V^{H} \end{array}\right]\;$$

Hence a projection matrix in the form of **P** in Equation (12) can be obtained as:

(22) $$P=\left[\begin{array}{cccccccc} V_{\left(1\right)}^{H}A^{H} & V_{\left(2\right)}^{H}A^{H} & \cdots & V_{\left(\frac{M}{2}\right)}^{H}A^{H} & V_{\left(\frac{M}{2}\right)}^{T}A^{H} & \cdots & V_{\left(2\right)}^{T}A^{H} & V_{\left(1\right)}^{T}A^{H} \end{array}\right]$$

where $V_{\left(i\right)},i=1,2,..\frac{M}{2}$ can be obtained by partitioning the eigenvector matrix **V** in Equation (21) associated to the interference-free subspace as:

(23) $$\underset{\frac{MN}{2}\times \left(\frac{MN}{2}-K\right)}{V}=\left[\begin{array}{c} \underset{N\times \left(\frac{MN}{2}-K\right)}{V_{\left(1\right)}}\\ V_{\left(2\right)}\\ \vdots \\ V_{\left(\frac{M}{2}\right)} \end{array}\right]\;$$

In the last step, **u** in Equation (10) is obtained to maximize the SNR for the projected signal. The problem of interest is finding the optimal combination of the columns of the projection matrix to satisfy the following relation:

(24) $$a=AP_{\left(i\right)}^{H}u,\;i=1,2,...,M$$

or equivalently:

(25) $$\vec{a}=P^{H}u$$

where $\vec{a}$ is defined as:

(26) $$\underset{MN\times 1}{\vec{a}}=\left[\begin{array}{c} a\\ a\\ \vdots \\ a \end{array}\right]$$

Taking Equations (9) and (10) into account, using Equation (24) to obtain **u** results in a constructive combination of the projected signal. From Equation (25), **u** can be obtained as:

(27) $$u=P\vec{a}$$

**u** can be also determined by allocating power to certain taps to partially control the shape of the resulting correlation function. This can be realized by changing the elements of $\vec{a}$ in Equation (25).

### 3.3. Steering Vector Estimator

In the previous section, it was assumed that the steering vectors are known. This section addresses the steering vector estimation from the output of the blind STP block. Herein, it is assumed that the array is calibrated which means constant uncertainties such as unequal cable lengths and coupling coefficients between antennas; also the antenna elements imperfections and their dependency on the signal AOA are assumed to be compensated [25]. Therefore for a calibrated array, the simplified baseband representation of the received signal in Equation (1) after despreading and projection can be written as:

(28) $$\underset{\left(MN-K\right)\times 1}{y}={U_{Null}}^{H}\vec{a}+n$$

in which $\vec{a}$ is defined in Equation (26) and **n** is the noise vector in the correlator outputs. During the tracking stage, the phase of the signal at one of the blind STP block outputs (reference output) is kept approximately zero and the phase of the signals at the other outputs are measured relative to that of the reference output (see Figure 2). For simplicity, amplitudes are also normalized with respect to the signal at the reference output. Therefore, the normalized signal vector that is actually measured from the multi-antenna receiver, denoted by $\tilde{y}$, is related to **y** as:

(29) $$\tilde{y}=\frac{y}{\delta^{T}{U_{Null}}^{H}\vec{a}}$$

in which $\underset{\left(MN-K\right)\times 1}{\delta }={\left[1,0,\cdots 0\right]}^{T}$ (without loss of generality, the first output of the blind STP block is chosen as the reference for despreading the signals of other outputs). Given Equations (29) and (28), $\vec{a}$ can be estimated using the following minimization problem:

(30) $$\underset{\begin{array}{l} \;\vec{a}\\ \;\left\|\vec{a}\right\|=1 \end{array}}{\min}\;E\left\{{\left\|\tilde{y}\delta^{T}{U_{Null}}^{H}\vec{a}-{U_{Null}}^{H}\vec{a}\right\|}^{2}\right\}$$

in which the constraint avoids the trivial solution that is $\vec{a}=0$. After some matrix manipulations, one can verify that $\vec{a}$ can be estimated from the following eigenvalue decomposition problem as:

(31) $$\underset{\begin{array}{l} \;\vec{a}\\ \;\left\|\vec{a}\right\|=1 \end{array}}{\min}\;{\vec{a}}^{H}U_{Null}D{U_{Null}}^{H}\vec{a}$$

where **D** is defined as:

(32) $$D=E\left\{{\left(\tilde{y}\delta^{T}-\underset{\left(MN-K\right)}{I}\right)}^{H}\left(\tilde{y}\delta^{T}-\underset{\left(MN-K\right)}{I}\right)\right\}$$

and **I** is the MN−K×MN−K identity matrix. Assume that $\vec{a}$ is written with respect to the parallel ($a_{∥}$) and orthogonal ($a_{⊥}$) components in the projected interference-free subspace as:

(33) $$\vec{a}=U_{Null}a_{∥}+U_{Int}a_{⊥}$$

By substituting Equation (33) in Equation (31), the optimization problem becomes:

(34) $$\underset{\left\|a_{∥}\right\|=1}{\underset{a_{∥}}{\min}}\;{a_{∥}}^{H}Da_{∥}$$

**D** has a rank deficiency of 1 and hence by SVD of **D**, $a_{∥}$ is obtained as the eigenvector corresponding to the smallest eigenvalue. As expected, $a_{⊥}$ cannot be estimated from the optimization problem since it is orthogonal to the projection matrix. For some satellite signals, $\left\|a_{∥}\right\|$ can be significant whereas for some of them, it is a small value. For better estimation of attitude parameters, only estimated steering vectors with larger values of $\left\|a_{∥}\right\|$ are employed. To measure how close the estimated steering vector ($\hat{a}$) is to the true steering vector, the following criterion is adopted:

(35) $$\;Cr=\left\|\hat{a}.{\hat{a}}^{*}-a.a^{*}\;\right\|=\left\|\hat{a}.{\hat{a}}^{*}-1\;\right\|\;$$

where (·) denotes the Hadamard product. This criterion simply determines how much the structure of the estimated $\hat{a}$ is similar to the structure of a typical steering vector defined in Equation (2). The value of *Cr* decreases if the structure of $\hat{a}$ becomes similar to the one defined in Equation (2) and becomes zero if $\hat{a}$ has the exact same structure.

### 3.4. Attitude Determination

Precise attitude determination is essential in many applications. Generally, differential carrier phase measurements from several receivers with precisely determined baselines or/and INS are employed for attitude determination. Since the proposed receiver is already equipped with an antenna array, herein beamforming using a calibrated antenna array is employed for attitude determination [26]. Moreover, in contrast to the standard attitude determination based on carrier phase measurements, in this method one GNSS signal along with an adequate number of antenna elements is enough for attitude determination. This is one of the main advantages of array-based attitude determination techniques, especially in interference environments where the number of available signals is still low after blind STP interference mitigation. It should be considered that the performance of the proposed method could become affected by multipath propagation. Herein, multipath propagation was not taken into account in the mathematical model of the proposed method. In [26], it was shown that for heading estimation in open sky conditions an approximate agreement of 1° with a SPAN GPS-INS system serving as reference was achieved; the experiment is repeated in interference environments here by applying the proposed receiver structure.

Considering Equation (2), **d***_l_* (a vector pointing to the *l*th satellite in the body frame coordinate system) can be derived as:

(36) $$d_{l}=\frac{\kappa }{2\pi }{\left(Z^{H}Z\right)}^{-1}Z^{H}∠{\hat{a}}_{l},\;l=1,2,...,L$$

in which ${\hat{a}}_{l}$ is a vector indicating the phase of the estimated steering vector elements of the *l*th satellite, L is the number of available satellites and **Z** is the array configuration matrix defined as:

(37) $$Z={\left[\begin{array}{cccc} z_{1} & z_{2} & \cdots & z_{N} \end{array}\right]}^{T}$$

Moreover, from the approximate position of the receiver and ephemeris information, this vector can be also expressed in the East-North-Up (ENU) coordinate system. Unit vectors pointing to all satellites expressed in the ENU and body frame coordinate systems are related to each other through:

(38) $$\begin{array}{l} E_{ENU}=R_{B}^{ENU}E^{B},\\ E^{B}=\left[\begin{array}{cccc} d_{1} & d_{2} & \cdots & d_{L} \end{array}\right] \end{array}$$

where $E_{ENU}$ is a matrix consists of satellite pointing vectors in the ENU coordinate systems and $R_{B}^{ENU}$ is the transformation matrix from the body frame to the ENU coordinate system [27]. It is convenient to express this transformation by using an Euler angles parameterization that has a direct physical interpretation. In this way, $R_{B}^{ENU}$ is formed based on three angles r, p and h referring to the roll, pitch and heading (yaw) angles and can be expressed as:

(39) $$R_{B}^{ENU}=\left[\begin{array}{ccc} \cos\left(h\right)\cos\left(r\right)-\sin\left(r\right)\sin\left(p\right)\sin\left(h\right) & -\sin\left(h\right)\cos\left(p\right) & \cos\left(h\right)\sin\left(r\right)+\sin\left(h\right)\sin\left(p\right)\cos\left(r\right)\\ \sin\left(h\right)\cos\left(r\right)+\sin\left(r\right)\sin\left(p\right)\cos\left(h\right) & \cos\left(h\right)\cos\left(p\right) & sin\left(h\right)\sin\left(r\right)-\cos\left(h\right)\sin\left(p\right)\cos\left(r\right)\\ -\sin\left(r\right)\cos\left(p\right) & \sin\left(p\right) & \cos\left(p\right)\cos\left(r\right) \end{array}\right]$$

For a given $E^{B}$ and $E_{ENU}$, estimating the transformation matrix $R_{B}^{ENU}$ from Equation (38) is equivalent to finding a solution to a least squares estimation problem. Therefore, by having attitude parameters, satellite azimuth and elevation angles, the steering vector of each satellite signal before blind projection can be accurately estimated even if it is not acquired or tracked after the blind STP filter. It is worth mentioning that due to the long distance between satellites and the receiver, azimuth and elevation angles can be estimated with enough accuracy even when degraded positioning performance occurs due to blind STP filtering.

## 4. Experimental Results

Due to frequency regulations, outdoor radio frequency (RF) power transmission in the GNSS frequency bands is prohibited. Therefore, special considerations have to be taken into account while testing the performance of anti-interference techniques. Some previous work has suggested combining interference signals to GNSS signals through wires. However, for an array antenna, this type of test requires many combiners, cables and connectors and moreover control on the angle of arrival of GNSS and interferer signals would be difficult. Herein for testing and evaluating the performance of the proposed method, interference has been generated in software and added to the digitized GNSS samples.

**Figure 3:**
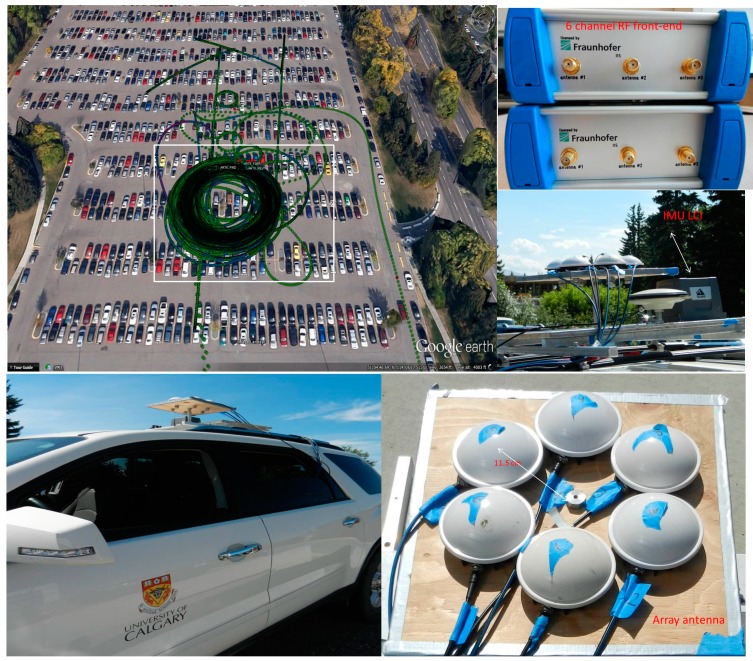
Data collection scenario and setup.

**Figure 4:**
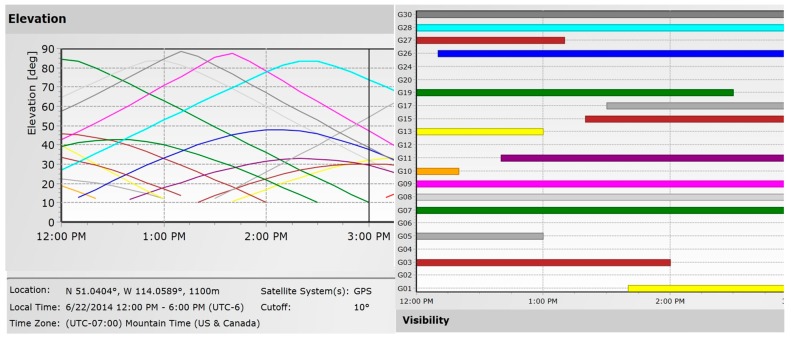
Availability and elevation angles of satellites during test (http://www.trimble.com).

The test set up and trajectory are shown in Figure 3; GPS L1 C/A signals were collected using an array of six antennas. The data collection was performed with an open view of satellites. A circular trajectory was driven for the calibration process to cover all azimuth angles. In order to cover a wide range of elevation angles, the circular trajectory was repeated several times over a two-hour interval. The data collection interval was long enough to allow satellites to move significantly in the sky to cover most elevation angles. Satellite visibility and elevation during the data collection are shown in Figure 4. Satellite Pseudo Random Noise (PRN) codes, elevation and azimuth angles used for the proposed space-time interference mitigation method are also shown in Table 1. The antenna array was mounted on the top of a vehicle and the six antenna elements were connected to a phase coherent six-channel Fraunhofer/TeleOrbit RF front-end. The received signals were then sampled, down converted and stored for post processing. Moreover, a NovAtel SPAN^TM^ LCI system, which includes a NovAtel SPAN^®^ enabled GNSS/INS receiver (SPAN SE) and a tactical grade IMU LCI was used as a reference to provide reference heading values and positions for comparison purpose. IMU measurements were sent to the receiver where a coupled GNSS/INS position, velocity and attitude solution was generated. Raw GPS data was also collected under Line of Sight (LOS) conditions using another receiver as a base station to provide differential positioning. The data collected by SPAN and the base station file were then fed to the NovAtel Inertial Explorer^®^ post-processing software to produce accurate reference orientation angles (the estimated standard deviations for position in each direction are below 5 cm and the estimated standard deviations for attitude parameters are below 0.1°).

The precise antenna array calibration method proposed in [25] was employed to calibrate the antenna array. In this method, a two-stage optimization for precise calibration is used in the form of two EVD problems. In the first stage, constant uncertainties are estimated whereas in the second stage the dependency of each antenna element gain and phase patterns to the received signal AOA is considered for refined calibration. An open source MATLAB-based single antenna software receiver [28] was modified as a multi-antenna receiver where the acquisition, tracking and position solution parts of the original software were modified. The interference mitigation units were added to the receiver and the structure of the receiver was changed from a “single-antenna tracking” to a “multi-antenna multi-delay tracking” receiver employing both spatial and temporal processing.

**Table 1:** PRNs used during test and corresponding azimuth and elevation angles and C/N_0_ after each interference mitigation stage.

PRN	Azimuth (degrees)	Elevation (degrees)	C/N_0_ Blind STP (dB-Hz)	C/N_0_ Distortionless STP (dB-Hz)
1	342	19	39.1	44.1
3	45	6	37.7	46.4
7	321	31	42.6	44.7
8	328	55	50.5	49.1
9	308	72	54.1	54.6
11	4	32	35.1	43.5
15	139	25	45.7	44.5
17	256	30	37.8	49.9
19	46	18	41.1	49.8
26	179	48	__	50.2
28	159	81	39.0	50.4
30	319	62	53.6	52.3

**Figure 5:**
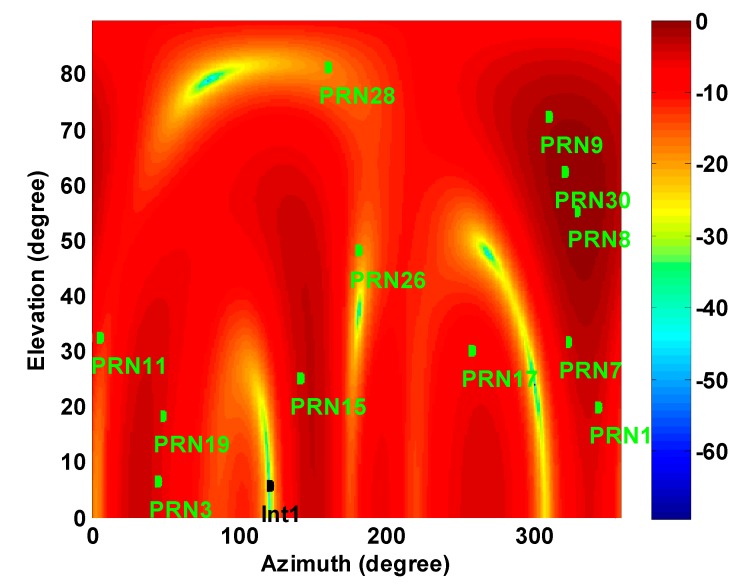
Normalized antenna array gain pattern at the interference frequency.

In the first test, one CW signal as an interference signal at GPS L1 centre frequency is added to the received signals. The elevation and azimuth of the interference signal are 7.5° and 120° and the interference-to-noise density ratio (I/N_0_) is 90 dB-Hz. The number of the TDL taps is 4. Figure 5 shows the array gain pattern after applying the blind projection matrix. As shown, a deep null is placed in the direction of the interference; however, since the steering vectors of the satellite signals have not been employed in the space-time filter structure, some of the desired signals are also unintentionally attenuated or even nulled out.

**Figure 6:**
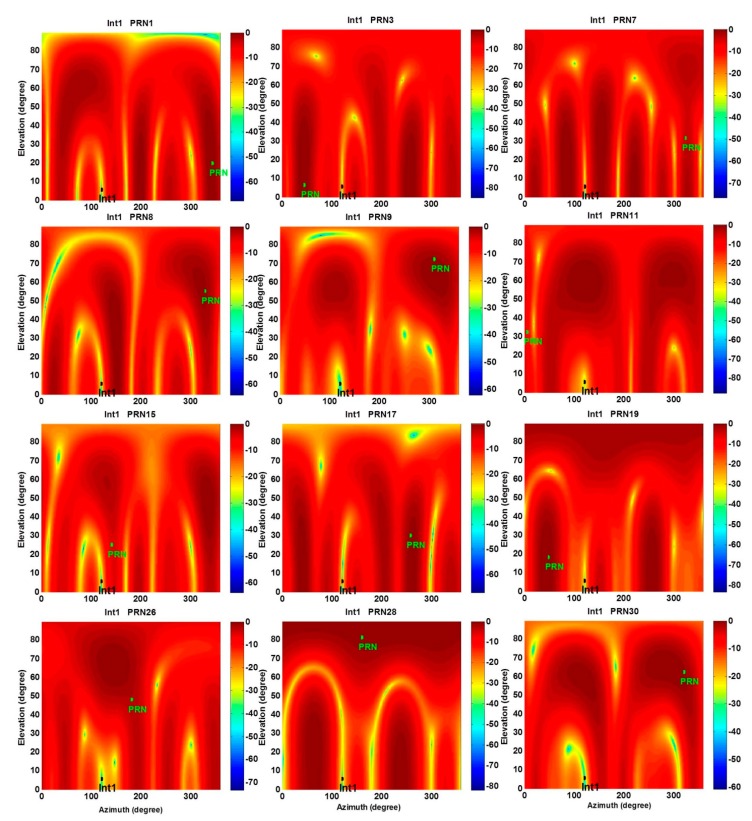
Normalized antenna array gain patterns at the interference frequency for the proposed distortionless STP filtering.

In the second stage of the proposed receiver and after employing the steering vectors, the STP filter coefficients can be determined to not only nullify the interference but also steer the main lobe of the array gain pattern into the direction of the desired signal as shown in Figure 6. Considering Equation (9), for a space-time filtering, the gain pattern (in dB) is calculated as $10\log\left({\left|h^{H}\left(f\right)a\right|}^{2}\right)$ where $h^{H}\left(f\right)a$ is a response of the filter to the impinging signal with the steering vector a and frequency *f*. In fact, this gain pattern determines the space-time filter gain at a specific frequency, azimuth and elevation angles.

**Figure 7:**
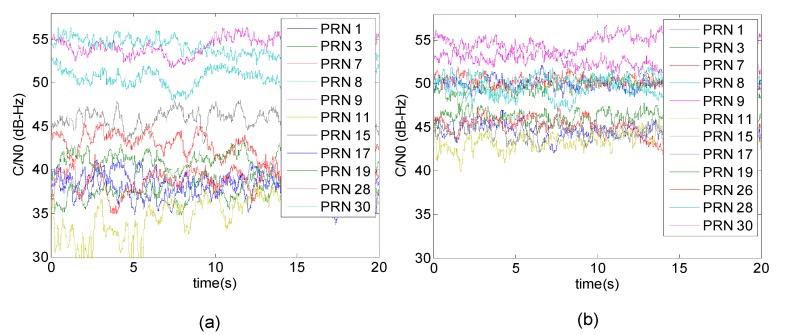
(**a**) Measured C/N_0_ after employing the blind STP filtering (**b**) and after employing the proposed distortionless STP filtering.

In order to obtain an actual sense of the improvement achieved, Figure 7 compares C/N_0_ values between blind and distortionless STP filtering for the same 20 s of received satellite signals. Average C/N_0_ values are also shown in Table 1. Since PRN 8, 9 30 are located close to the main lobe of the gain pattern in the blind filter, after applying the distortionless filter, their C/N_0_ values are slightly decreased or not considerably changed. This is due to the fact that the DoF is halved to maintain linearity of the phase response for the distortionless filter. However, the average C/N_0_ of all PRNs is increased approximately by 5 dB-Hz (up to 12 dB-Hz for PRNs 28 and 17). In addition, PRN 26 was significantly attenuated and denied after blind filtering but it could be acquired and tracked by employing the distortionless STP filtering.

The performance of the attitude determination block is now reported. Recall that the SPAN LCI system was used as a reference to provide external and independent heading and attitude parameters. Assuming a horizontal motion and considering Equations (38) and (39), the heading angle can be estimated using the following simplified relation:

(40) $$E_{ENU}=\left[\begin{array}{ccc} \cos\left(h\right) & \sin\left(h\right) & 0\\ -\sin\left(h\right) & \cos\left(h\right) & 0\\ 0 & 0 & 1 \end{array}\right]E^{B}$$

Based on the criterion in Equation (35), PRNs 30, 9 and 8 are chosen for heading determination. In fact, as shown in Figure 5, these PRNs are located close to the main lobe of the array gain pattern. Therefore, employing these signals leads to more accurate estimates of steering vectors and consequently heading angles. In this test, heading angles were calculated within a 40 s interval in static mode.

**Figure 8:**
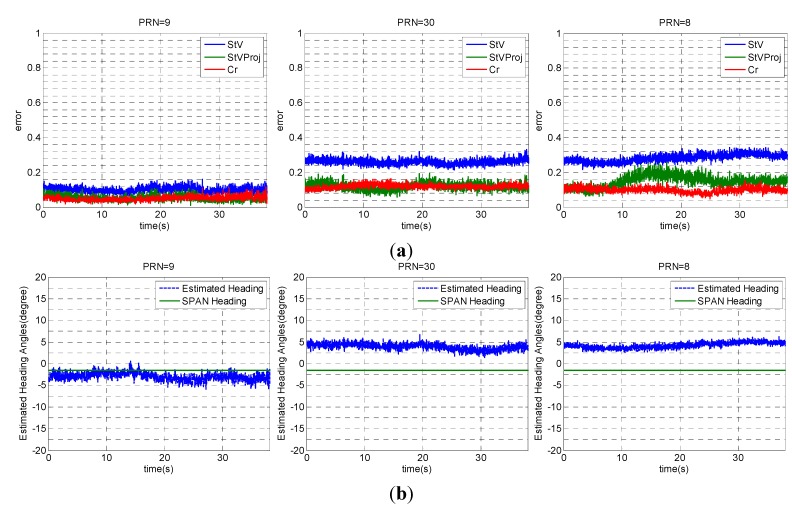
(**a**) Comparison of estimated steering vectors (**b**) and estimated heading angles for PRN 9, 30 and 8.

Figure 8a displays two error types. The first error (StV) compares the estimated steering vector from the proposed receiver $\left(\hat{a}\right)$ with that measured from the SPAN system (a) and the second error (StVProj) compares $\hat{a}$ with the parallel projected component of the measured steering vector by the SPAN system ($a_{∥}$). These errors are calculated as:

(41) $$\begin{array}{l} StV\;error=\frac{\left\|\hat{a}-a\right\|}{\left\|a\right\|}\\ StV\mathrm{Proj}\;error=\frac{\left\|\hat{a}-a_{∥}\right\|}{\left\|a_{∥}\right\|} \end{array}$$

**Figure 9:**
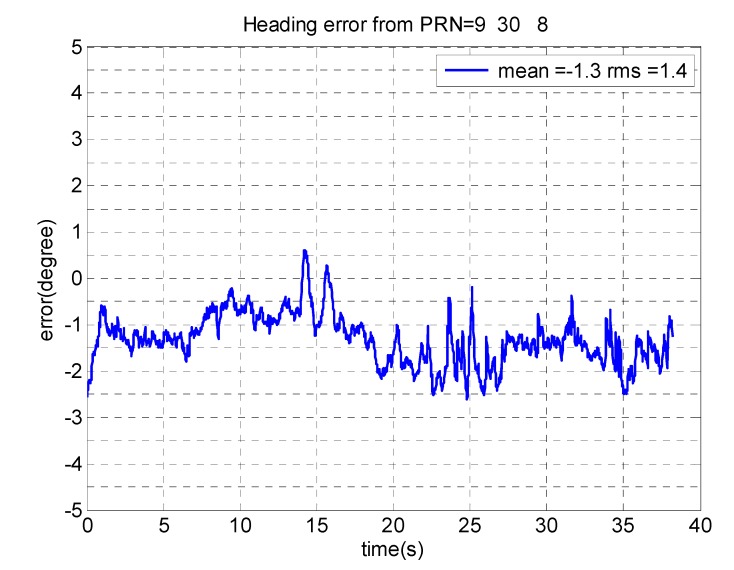
Error in heading angles obtained from PRNs 9, 30 and 8.

Results show that the StVProj errors are less than the StV errors, which verifies the fact that $\hat{a}$ is an estimate of the parallel component $a_{∥}$, and the orthogonal component $a_{⊥}$ cannot be estimated (see Equations (33) and (34)). Figure 8a also shows the value of *Cr* calculated in Equation (35), which reveals that the structure of estimated steering vectors is close to that of a true steering vector as defined in Equation (2). Figure 8b compares the estimated heading angles for each of these three PRNs with those obtained with the SPAN system. Figure 9 shows the errors of the estimated heading angles considering all these PRNs, showing an approximate agreement of 1.5° for heading estimates. In practice and in a dynamic operation environment, a receiver should constantly select among satellite signals with lower *Cr* values for estimating attitude parameters. Selecting proper satellite signals also depends on their elevation angles. In fact, satellites with higher elevation angles show poorer accuracy for heading estimation such that the satellite located at the receiver’s zenith cannot be used to sense the horizontal motion. However, the errors due to multipath and noise are higher for satellites with low elevation angles. Therefore, mid elevation satellites could be a good option for extracting attitude parameters.

As mentioned before, the blind STP may distort the correlation functions and produce biases in the pseudorange measurements. Moreover, some satellites may be unintentionally nullified, which in turn reduces the Dilution of Precision (DOP). These biases and resulting attenuations degrade the position solution accuracy. Table 2 lists errors in the ENU coordinate system for three interference scenarios for distortionless and blind filters.

**Table 2:** Results for different interference scenarios.

20 s of data in the static mode		Scenario 1 One CW Interfernce	Scenario 2 One CW & One Wideband Interfernce	Scenario 3 Six CW Interfernce
		I/N_0_ = 90 dB-Hz TDL = 4	I/N_0_ = 90 dB-Hz TDL = 4	I/N_0_ = 90 dB-Hz TDL = 6
Blind STP ENU error (m)	E (mean,rms)	(−0.8,0.9)	(−4.0,4.8)	~(500,500)
	N (mean,rms)	(−0.7,1.3)	(−8.1,9.6)	~(1000,1000)
	U (mean,rms)	(−3.9,4.1)	(−8.0,8.2)	~(1000,1000)
MPDR ENU error (m)	E (mean,rms)	(−0.6,0.6)	(−0.8,1.0)	-
	N (mean,rms)	(−0.9,1.0)	(−1.2,1.3)	-
	U (mean,rms)	(−1.9,2.0)	(−2.5,2.7)	-
STP MPDR ENU error (m)	E (mean,rms)	(−0.8,0.9)	(−3.5,3.6)	(−70.2,75.0)
	N (mean,rms)	(−1.7,1.8)	(−4.0,4.1)	(116.9,126.5)
	U (mean,rms)	(−8.4,8.4)	(−13.5,13.6)	(−148.8,149.2)
Proposed Distortionless STP ENU error (m)	E (mean,rms)	(−0.4,0.6)	(−1.2,1.5)	(−0.1,0.3)
	N (mean,rms)	(−0.4,0.5)	(−0.6,0.7)	(−0.5,0.6)
	U (mean,rms)	(−0.2,1.0)	(−0.5,0.7)	(2.7,2.7)
Blind STP Average C/N_0_ (dB-Hz)		43.3	44	41.9
MPDR Average C/N_0_ (dB-Hz)		51.4	49.6	-
STP MPDR Average C/N_0_ (dB-Hz)		51.4	50.0	46.2
Proposed Distortionless STP Average C/N_0_ (dB-Hz)		48.3	44.2	45.7
Blind STP Number of PRNs acquired & tracked		11	8	5
MPDR Number of PRNs acquired& tracked		12	12	-
Proposed Distortionless STP & STP MPDR Number of PRNs acquired& tracked		12	12	12

The I/N_0_ for each wideband or narrowband signal is 90 dB-Hz. The interference signals are spread over the GPS L1 frequency band and have incident elevation angles in the range of 2° to 10°. In this table, the number of acquired and tracked satellites after employing the proposed blind and distortionless STP filters and the resulting average C/N_0_ are also shown. In the simple interference scenario (Scenario 1), the number of acquired satellite signals is slightly different between blind and distortionless STPs and the resulting improvement in position solution is due to decreasing the distortion and bias on correlation functions; in the harsh interference scenario (Scenario 3), improvement occurs also because of increasing the number of acquired satellites and DOP improvement. In general the improvement obtained with the proposed methods depends on the interference scenario, antenna array configuration, number of taps in TDLs, calibration accuracy, power and direction of GNSS and interference signals, DOP and other factors which have not been analyzed in this paper.

In order to highlight the advantages of the purposed distortionless STP filter, its performance is compared to the conventional MPDR (space only processing) and modified STP MPDR beamformers briefly introduced as follows:

*MPDR Beamformer:* this beamformer has been widely employed in array based applications. In GNSS applications, as long as the array is calibrated and its orientation is determined, MPDR is one of the powerful approaches available to suppress interfering signals while maintaining desired signals. The optimization problem for the MPDR beamformer can be expressed as:

(42) $$\begin{array}{l} \underset{\vec{w}}{Min}\;w^{H}Rw\\ \;a^{H}w\text{=}1 \end{array}$$

where R is the spatial correlation matrix, a is the steering vector defined in Equation (2) and w is the array weighting vector. The goal is to minimize the power subject to the constraint. This minimization problem can be solved by using a Lagrange multiplier approach. The optimal gain vector is obtained as [5]

(43) $$w=R^{-1}a{\left(a^{H}R^{-1}a\right)}^{-1}$$

*STP MPDR* Beamformer*:* this beamformer is the extended version of the MPDR beamformer for space–time processing and also employed in several papers (e.g., [18,19,20]). The degree of freedom of the array compared to MPDR beamformer is increased. The optimization problem for the MPDR beamformer can be expressed as:

(44) $$\begin{array}{l} \underset{\vec{w}}{Min}\;{\vec{w}}^{H}R_{\vec{r}}\vec{w}\\ \;c^{H}\vec{w}\text{=}1 \end{array}$$

where $R_{\vec{r}}$ and $\vec{w}$ are defined in Equations (4) and (6) and the vector  c is defined as:

(45) $$\underset{NK\times 1}{c}={\left[\begin{array}{cccc} a^{T} & \underset{N\times 1}{0^{T}} & \cdots & \underset{N\times 1}{0^{T}} \end{array}\right]}^{T}$$

In this filter, in order to force the beamformer to have a fixed group delay, only one group of tap gains with a certain delay (without loss of generality here the first group is chosen) is required to pass the satellite signal undistorted. However, the filter response is not necessarily linear in phase and cross correlation functions may be asymmetrical and distorted. This minimization problem can be solved using a Lagrange multiplier approach.

For the MPDR beamformer, the array DoF, indicating the number of unwanted signals that can be nullified, is equal to the number of antenna elements minus one. Since this beamformer is only based on spatial processing, cross correlation functions and position solutions do not experience any distortion due to time filtering. ENU error results for this beamformer, reported in Table 2, verify the fact that the MPDR beamformer can suppress the first two scenarios without generating significant ENU errors but is not able to mitigate six uncorrelated narrowband interference signals. However, the proposed distortionless STP filter not only provides extra DoF for narrowband interference mitigation but also keeps the cross correlation functions undistorted. The results also show that the proposed STP filter outperforms the STP MPDR beamformer in terms of distortions and ENU errors. Contrary to the STP MPDR beamformer, the proposed STP filter is designed not only to maximize the SNR but also to be linear in phase. In other words, the proposed STP filter is spatially and temporally distortionless. It should be noted that for the proposed STP filter the resulting average C/N_0_ values are slightly lower than those of the space-only and STP MPDR beamformers because of applying a restriction on the structure of the filter. However, as shown in Table 2, the positioning performance of the proposed approach considerably outperforms that of the STP MPDR method.

## 5. Conclusions

An array-based GNSS receiver capable of interference mitigation and attitude determination was proposed. Two types of space-time filtering were employed in the structure of the receiver, namely blind and distortionless STP methods. The blind STP block projects the received signals into the reduced rank interference-removed subspace without any consideration of GNSS distortion, bias and attenuation. It was shown that the array attitude parameters could be estimated from the output of the blind STP block and this information were then employed to calculate the steering vector of all available satellite signals. Using the calculated steering vectors, the proposed distortionless STP block was able to assign specific filter coefficients to the signal of each satellite and shape the array gain pattern to simultaneously nullify the interference signals and put the main lobe toward the direction of each desired GNSS satellite while maintaining the filter phase response linear. Simulation and experimental results showed that the proposed distortionless STP method acquired and tracked a higher number of satellites compared to the blind STP. Moreover, the distortionless STP resulted in less distortion and bias on pseudorange measurements and consequently provided more accurate position and timing solutions (the improvement of a few metres in ENU errors in the simple interference scenario to a few hundred metres in a challenging one). Experimental results also showed that the positioning performance of the proposed approach is considerably higher than that of the STP MPDR method. The proposed STP receiver structure removes the need for a priori knowledge of the antenna array attitude since it is able to accurately estimate it using available GNSS satellites. The results reported here were limited to a few specific cases to show the applicability of the proposed receiver structure. The next step could be the design of an approach to adaptively nullify interference signals to allow the proposed receiver to operate in dynamic mode.
